# Drugs responsible of DRESS syndrome regulate IL-10 and TNF-α secretion

**DOI:** 10.1186/2045-7022-4-S3-P45

**Published:** 2014-07-18

**Authors:** Sébastien Calbo, Bassam Sabbah, Baptiste Janela, Damien Picard, Maud Maho-Vaillant, Pascal Joly, Philippe Musette

**Affiliations:** 1 Inserm U905, Normandie Univ, France; 2 Singapore Immunology Network, A STAR, Singapore

## Background

Drug reaction with eosinophilia and systemic symptoms (DRESS) is a severe drug-induced reaction that involves both the skin and the viscera. Several herpesvirus family members like EBV or HHV-6 can be detected coincidently with various clinical symptoms in DRESS. In addition, we have previously identified activated EBV specific cytotoxic CD8+ T cells as major actors in the pathophysiology of DRESS. In vitro, we have showed that DRESS inducer drugs increase production of EBV virus only on B-LCL lines from DRESS patients. However, drug effect on cytokines secretion has not been studied. Gene expression profiling of DRESS patients' PBMC revealed that IL-10 and TNF-α were two of the most upregulated mRNA. We thus measured IL-10 and TNF-α secretion levels in DRESS patients' serum and B-LCL lines following incubation with drugs.

## Method

EBV-B cell lines were obtained after incubation of B cells from DRESS patients or healthy donors with EBV virus. Also, DRESS patients and healthy donors PBMC and serum were included in the study. We analysed the presence of IL-10 by ELISA, FACS and QPCR, and the presence of TNF-α by ELISA and FACS.

## Results

We show that DRESS patients have an increase of IL-10 and TNF-α in their serum. IL-10 is not secreted by CD4+ T cells but DRESS patients have regulatory B cells which, under stimulation, produce two times more IL-10 than B cells from healthy controls. In vitro, we demonstrate that some DRESS inducer drugs reduce significantly the IL-10 secretion in B-LCL from DRESS patients but not from healthy donors by sequestering IL-10. Interestingly, the same observation was obtained for TNF-α in DRESS patients and healthy donors B-LCL, with however differential effect depending of the drugs regarding the sequestering of TNF-α.

## Conclusion

the balance between IL-10 and TNF-α is affected by DRESS inducer drugs specifically in DRESS patient. These findings allow a better understanding of the physiopathology of the DRESS syndrome.

